# Travel from the United Kingdom to the United States by a Symptomatic Patient Infected with the SARS-CoV-2 B.1.1.7 Variant — Texas, January 2021

**DOI:** 10.15585/mmwr.mm7010e2

**Published:** 2021-03-12

**Authors:** Moriam Ojelade, Annette Rodriguez, Dante Gonzalez, Denzel Otokunrin, Srikanth Ramachandruni, Elizabeth Cuevas, Kelly Moon, Carla Gutiérrez Tyler, Melissa Freeland, Mark Anderson, Kambria Haire, Yuridia Orozco, Fija Scipio, Yuri Springer, Emilie Prot, Jennifer A. Shuford

**Affiliations:** ^1^Corpus Christi–Nueces County Public Health District, Corpus Christi, Texas; ^2^Texas Department of State Health Services; ^3^CDC COVID-19 Response Team; ^4^Division of Global Migration and Quarantine, National Center for Emerging and Zoonotic Infectious Diseases, CDC.

In December 2020, the B.1.1.7 genetic variant of SARS-CoV-2, the virus that causes COVID-19, was first reported after emergence and rapid circulation in the United Kingdom (*1*). Evidence suggests that the B.1.1.7 variant is more efficiently transmitted than are other SARS-CoV-2 variants, and widespread circulation could thereby increase SARS-CoV-2 infection and hospitalization rates (*1*,*2*). The first reported SARS-CoV-2 B.1.1.7 variant case in the United States was confirmed by sequencing in Colorado on December 29, 2020.* This report describes a person who traveled from the United Kingdom to the United States after experiencing COVID-19–compatible symptoms^†^ and was eventually confirmed to be infected with the B.1.1.7 variant.

On January 10, 2021, CDC notified the Texas Department of State Health Services (DSHS) of a SARS-CoV-2 B.1.1.7 variant case; Corpus Christi–Nueces County Public Health District staff members conducted a case investigation on January 10–11. The patient, aged 61 years, had visited family in the United Kingdom during November 13–December 30, 2020, and reported having been exposed to a relative experiencing COVID-19–compatible symptoms (cough, runny nose, and headache) on December 24. Another relative at the same gathering received a positive COVID-19 test result in the United Kingdom on January 10. The patient received a negative SARS-CoV-2 antigen test result on December 28 in preparation for travel back to the United States but experienced symptoms on December 29 and reported taking acetaminophen on December 30. On December 30, the patient disclosed a runny nose during the pretravel interview but was cleared to fly from London to Dallas, Texas the same day. Upon arrival in the United States on December 31, the patient stayed overnight in a hotel and then drove home (approximately 8 hours). On the way home, the patient stopped five times, including twice for food, twice for gas, and once at a grocery store. Throughout the international and domestic travel period, the patient reported trying to maintain physical distance from others and wearing a cloth face mask, except while eating or drinking. The patient began self-quarantine upon returning home, which was broken twice for a medical and testing appointment. Additional symptoms, including loss of taste and smell, severe headache, chills, and a dry cough, began on January 1. On January 2, the patient sought confirmation of SARS-CoV-2 infection by real-time reverse transcription–polymerase chain reaction (RT-PCR) testing and received a positive test result on January 4, at which point the patient began a 10-day isolation. The RT-PCR exhibited S-gene target failure, a diagnostic test result suggestive of the B.1.1.7 variant (*2*). This finding was confirmed by sequencing at a commercial laboratory affiliated with CDC’s national strain surveillance system.^§^

As part of the contact investigation, Texas DSHS shared the patient’s flight information with the CDC El Paso Quarantine Station on January 11. Because 12 days had passed since the flight, CDC did not initiate an aircraft contact investigation; however, CDC later provided an informational notification to the states because of the variant case. The patient’s single asymptomatic pediatric household contact was not tested but quarantined concurrently with the patient. No secondary cases with epidemiologic links to the patient have been identified to date.

This case demonstrates how a variant of concern, in this case B.1.1.7, might be translocated between communities through travel. At the time of this person’s travel, CDC had an order in place requiring proof of a negative SARS-CoV-2 test ≤3 days before departure, or documentation of recovery from COVID-19, for all air passengers boarding a flight to the United States from the United Kingdom (*3*). Subsequently, on January 12, CDC issued an order expanding this requirement to all international air passengers arriving in the United States, effective January 26, 2021 (*4*). Because of the lower sensitivity of some SARS-CoV-2 antigen tests (*5*,*6*), the potential for false-negative results when nucleic acid amplification tests (such as RT-PCR) are administered shortly after infection with SARS-CoV-2 (*7*), and the subsequent potential for exposing others after a test is administered, predeparture testing should be considered one component of a comprehensive travel risk management strategy. Properly timed testing, both before and after travel, together with self-monitoring for symptoms, a period of self-quarantine after travel, use of a well-fitting mask, hand hygiene, and physical distancing, are critical elements of this strategy (*8*). Persons should not travel if they are experiencing symptoms compatible with COVID-19 or if they have received a positive SARS-CoV-2 test result and have not met criteria to discontinue isolation,^¶^ have had close contact with a person with suspected or confirmed COVID-19 and have not subsequently met criteria to end quarantine,** or have a pending SARS-CoV-2 viral test result.

## Footnotes

*
https://covid19.colorado.gov/press-release/cdphe-confirms-two-additional-cases-and-one-possible-case-of-the-b117-variant

† Patient initially experienced runny nose and headache before departure and later experienced loss of taste and smell, severe headache, chills, and a dry cough after return to the United States, which are symptoms compatible with COVID-19. https://www.cdc.gov/coronavirus/2019-ncov/symptoms-testing/symptoms.html; https://www.cdc.gov/flu/symptoms/flu-vs-covid19.htm

§
https://www.aphl.org/programs/preparedness/Crisis-Management/COVID-19-Response/Pages/Sequence-Based-Surveillance-Submission.aspx

¶
https://www.cdc.gov/coronavirus/2019-ncov/hcp/disposition-in-home-patients.html

**
https://www.cdc.gov/coronavirus/2019-ncov/more/scientific-brief-options-to-reduce-quarantine.html
